# Effect of ciliary neurotrophic factor on neural differentiation of stem cells of human exfoliated deciduous teeth

**DOI:** 10.1186/s13036-020-00251-4

**Published:** 2020-12-09

**Authors:** Sujuan Zeng, Xuedan Zhao, Lingling Zhang, Janak L. Pathak, Wenyan Huang, Yunyang Li, Hongbing Guan, Wanghong Zhao, Lihong Ge, Yan Shu

**Affiliations:** 1 Department of Pediatric Dentistry, Affiliated Stomatology Hospital of Guangzhou Medical University, Guangzhou Key Laboratory of Basic and Applied Research of Oral Regenerative Medicine, Guangzhou, 510182 China; 2 GuangDong Second Traditional Chinese Medicine Hospital, Guangzhou, 510095 China; 3 Department of Stomatology, Nanfang Hospital, Southern Medical University, Guangzhou, 510515 China; 4 Department of Pharmaceutical Sciences, School of Pharmacy, University of Maryland at Baltimore, Baltimore, MD USA

**Keywords:** Ciliary neurotrophic factor, Stem cells of human exfoliated deciduous teeth, Neurogenic differentiation, Cholinergic neuron

## Abstract

The stem cells of human exfoliated deciduous teeth (SHEDs) are considered to be one of the main sources of seed cells in stem cell therapy. The aim of this study was to examine the effect of ciliary neurotrophic factor (CNTF) on neurogenic differentiation of SHEDs. With the consent of parents, SHEDs from 6 to 8 year old children were isolated and cultured. The mesenchymal stemness and the potential of multidirectional (adipogenic and osteogenic) differentiation for the isolated SHEDs were firstly determined. The effect of CNTF on specific neurogenic differentiation of SHEDs was then examined by detecting the expression of marker genes and proteins via RT-PCR, immunoblotting, and immunofluorescence microscopy. The isolated SHEDs expressed specific surface markers of mesenchymal stem cells, and their potential of osteogenic and adipogenic differentiation were confirmed. CNTF promoted the differentiation of SHEDs into neuron-like cells with a high expression of acetylcholine transferase (CHAT), a marker of cholinergic neurons. The expression of other neuron markers including nestin, microtubule-associated protein 2 (MAP 2), and β-tublin III was also detected. Interestingly, the expression of neurogenic markers was maintained at a high level after neurogenic induction. SHEDs can be induced by CNTF to differentiate into cholinergic neuron-like cells under appropriate culture conditions. Our findings have laid a foundation for future use of SHEDs to treat neurological diseases.

**Supplementary Information:**

The online version contains supplementary material available at 10.1186/s13036-020-00251-4.

## Introduction

Stem cells are those with the potential to differentiate into different types of cells in the body. Growth factors can regulate the differentiation of stem cells. With appropriate growth factors and culture conditions, stem cells have been widely explored as seed cells in cell therapy which is used to replace the cells that are dysfunctional or dead, thereby restoring certain body function [1]. For example, bone marrow transplantation is the most widely used stem cell therapy, and cord blood-derived stem cells are also used in some treatments [2]. Choosing suitable stem cells and inducing them to become target cells, tissues, and organs is the key to successful stem cell therapy.

Many types of human stem cells have been derived from oral cavity, including dental pulp stem cells (DPSCs), stem cells of human exfoliated deciduous teeth (SHEDs), apical papillary stem cells, and periodontal ligament stem cells [3]. These stem cells are all mesenchymal origin. Among them, SHEDs have been found to possess the capability of self-renewal and multi-directional differentiation. DPSCs and SHEDs originate from migrating cranial neural crest (CNC) cells [4]. Since SHEDs are isolated form pediatric tooth, they could have higher stemness compared to adult dental pulp stem cells [5]. In particular, the neuronal differentiation capability of SHEDs has been reported to be stronger than those of DPSCs and bone marrow stem cells (BMSCs) [6]. In addition, SHEDs have immunomodulatory and anti-inflammatory effects [7]. Previous studies have shown that SHEDs can differentiate into multiple types of neurons, such as spiral ganglion neuron, dopamine neuron, photoreceptor-like cells [8–10]. After transplantation, SHEDs can even survive in mouse brain for more than 10 days after being induced in neuronal conditioning medium. The differentiated SHEDs can continue to divide and proliferate in mouse brain [11]. Protocols have been developed to promote the differentiation of stem cells including SHEDs into neuron-like cells [12, 13]. These studies have established the neurogenic differentiation potential of SHEDs. Given that SHEDs are easily available from the clinic with minimal ethical concerns, the potential of SHEDs in stem cell therapy for neurological and other diseases is of particular interest.

Ciliary neurotrophic factor (CNTF) was originally isolated from the ciliary ganglion of birds [14]. CNTF can promote the survival of different neurons and the differentiation of neural progenitor cells into astrocytes in vitro [15]. CNTF is a member of the interleukin-6 cytokine family [16]. It is mainly expressed in peripheral nervous system and the astrocytes of central nervous system. CNTF can be released after nerve injury to promote neuronal survival and regeneration [17]. It has been reported that CNTF can induce the differentiation of murine BMSCs into cholinergic neurons [18]. CNTF together with salvia extracts could promote the differentiation of myogenic stem cells into neuron-like cells in vitro [19]*.* In addition, CNTF has been reported to increase the expression of hippocampal acetyltransferase in diabetic rats and enhance their cognitive function [20]. A large number of studies have indicated an important role of CNTF in the proliferation and function of cholinergic neurons. Due to their ability of inducing the differentiation of certain stem cells into cholinergic neurons, CNTF and interleukin-6 are known as cholinergic factors [21].

Cholinergic neurons contain and releases acetylcholine [22]. Studies have shown that cholinergic neurons in the cortex and other brain regions may be involved in the regulation of complicate functions such as sleep, motor function, aggressive behavior, especially learning and memory [23, 24]. At present, the effect of CNTF on the differentiation of the stem cells from oral cavity including SHEDs is unknown. It has been reported that SHEDs are able to differentiate into dopaminergic neurons [13, 25, 26]. However, little information is available with respect to the differentiation of SHEDs into cholinergic neurons. In this study, we investigated the effect of CNTF on neural differentiation of SHEDs. In particular, we assessed the potential differentiation of SHEDs into cholinergic neuron-like cells in the presence of CNTF.

## Materials and methods

### Isolation, culture and identification of SHEDs

The primary teeth were all collected from the child patients from the Department of Children’s Stomatology, the Affiliated Stomatology Hospital, Guangzhou Medical University (KY2019008). The written and informed consents were obtained from the parents and the study was approved by the Research Ethics Committee of Guangzhou Medical University. The inclusion criteria for the primary tooth were: 6–8 years old, lower primary tooth, root resorption less than 1/3, no dental cavities, and no periapical disease. There was no history of systemic and hereditary diseases in the child donors.

The deciduous tooth and the peripheral area were thoroughly disinfected with 1% iodine tincture before extraction. After extraction, the tooth was washed with saline for 2 times, then put in 4 °C DEME medium containing 5 times penicillin/streptomycin for up to 4 h before SHED isolation. SHEDs were isolated according to the method described previously by Miura et al. [6]

SHEDs are stem cells of mesenchymal origin and possess the potential of adipogenesis and osteogenesis [27]. The identity of mesenchymal origin and differential potential of adipogenesis and osteogenesis for the isolated SHEDs were confirmed by flow cytometry, Oil red O staining, and Alizarin red staining, respectively, as described below.

### Flow cytometry analysis

The SHEDs of third passage (P) were labeled with fluorescein isothiocyanate-conjugated orphycoerythrin-conjugated antibodies and analyzed with flow cytometry. Cell aliquots (2.0 × 10^6^ cells) were incubated for 0.5 h at 4 °C with monoclonal antibodies specific for human CD34, CD45, CD90, CD105 (BD Biosciences), or isotype-matched control IgGs (Southern Biotechnology Associates). After washed with stain buffer for two times, the cells were incubated with second antibody at 4 °C for another 0.5 h. The expression profiles for the cell surface markers were analyzed by flow cytometry (Calibur; BD Biosciences).

### Alizarin red staining

P3 SHEDs were seeded at a density of 2.0 × 10^5^ cells/well into 6-well plates with routine DEME. When the cells become 80% confluence, the culture medium was replaced with osteogenic medium (Mesenchymal Stem Cell Osteogenesis Kit, Chemicon, USA). SHEDs were grown in the osteogenic medium for 14 days. To detect mineralization, the cells were fixed with 70% ethanol and stained with 2% Alizarin red (Sigma-Aldrich). The Alizarin red-positive cells were analyzed under microscopy by computer as described previously [17]. Osteogenesis was determined by cellular accumulation of Alizarin red-stained calcium.

### Oil red O staining

P3 SHEDs were seeded at a density of 2.0 × 10^5^ cells/well into 6-well plates with routine DEME. When the cells become 80% confluence, the culture medium was replaced with adipogenic medium (Mesenchymal Stem Cell Adipogenesis Kit, Chemicon, USA). SHEDs were grown in the adipogenic medium for 21 days. The cells were fixed with 4% formalin for 10 min at room temperature. After washed by PBS and incubated by isopropanol, the cells were stained with working solution of the Oil Red O for 30 min. The Oil Red O-positive cells were analyzed under microscopy by computer as described previously [28]. Adipogenesis was determined by lipid accumulation in fat vacuoles.

### Neurogenic differentiation of SHEDs induced by CNTF

P3 SHEDs were seeded at a density of 5.0 × 10^4^ cells/well into 6-well plates with routine DEME. When the cells become 80% confluence, the culture medium was replaced with neurogenic medium for mesenchymal stem cells (MSCs) (PromoCell, Germany) with and without addition of CNTF (5–20 ng/L). Preliminary studies were conducted to optimize the concentration of CNTF, and 15 ng/L of CNTF was chosen, unless otherwise indicated, based on its effects on the expression of marker genes and proteins as described below. The culture medium was changed every 2 days according to the instruction by PromoCell for the neurogenic medium for MSCs. The morphological changes of cells were observed and recorded under microscope. The neurogenic differentiation of SHEDs was determined by detecting the expression of Nestin, the neuron tubulin related marker MAP-2, β-tublin III, and the special cholinergic neuron surface marker CHAT, using qRT-PCR, immunoblotting, and immunofluorescence microscopy as described below.

### Quantitative reverse transcriptase polymerase chain reaction (qRT-PCR)

At 1, 2, 7, 14 and 21 days after neurogenic induction for SHEDs, qRT-PCR was used to detect the gene expression of β–tubulin III, MAP 2, nestin, and ChAT. Primer5.0 software was used to design the primers (Table 1). The primers were synthesized by Shanghai Terri Biological Company. qRT-PCR was performed in triplicate using SYBR Green (Applied Biosystems, Foster City, CA, USA) in a Real-Time PCR System 7500 (Applied Biosystems). Thermal cycling conditions included a 5-min step at 95 °C, followed by 95 °C for 10 s and 60 °C for 33 s. These steps were repeated for 40 cycles with melting curve analysis. Relative quantification of each gene was calculated after normalization to GAPDH housekeeping gene by using the 2^-ΔΔCT^ method.

**Table 1:** Primers used in qRT-PCR to examine gene expression

Genes	Primer Sequence	
	Forward	Reverse
*TUBB3*	5′ AACCAGATAGGGGCCAAGTT 3’	5′ GGCCTGAATAGGTGTCCAAA 3’
*MAP 2*	5′ ACCAACCACTGCCAGACCT 3’	5′ GTGGCGGATGTTCTTCAGAG 3’
*NES*	5′ AGCCCTGACCACTCCAGTTTAG 3′	5′ CCCTCTATGGCTGTTTCTTTCTCT 3’
*CHAT*	5′ ATGGCCATTGACAACCATCTTCTG 3’	5, AACAAGGCTCGCTCCCACAGCTTC 3’
*GAPDH*	5′ GGACCTGACCTGCCGTCTAG 3’	5′ GTAGCCCAGGATGCCCTTGA 3’

### Western immunoblotting

The cells were rinsed with PBS for 2 times. The cells were then digested with trypsin for 3 min. The cell suspension was centrifuged for 5 min, and the supernatant was discarded. Lysis buffer (10 mM N-(2-hydroxyethyl) piperazine-N-ethanesulfonic acid, 100 μM ethylenediaminetetraacetic acid (EDTA), 10 mM KCl, 0.5% NP40, protease and phosphatase inhibitor cocktails) was added to the cellular pellet. Cells were lysed for 20 min, vortexed for 10 s, and centrifuged at 13,000 g for 15 min. Proteins were quantitated with the bicinchoninic acid assay. The adjusted protein concentrations were resolved on 10% sodium dodecyl sulfate-polyacrylamide gel electrophoresis gel which was transferred to nitrocellulose membrane, and Western blot was carried out using antibodies specific for the proteins as followed: GAPDH, nestin, β–tubulin III, MAP 2, and ChAT (Abcam, England).

### Immunocytochemistry

Poly-lysine covered coverslips were placed in 24-well plates for SHED culture. After 80% confluence, the cells were cultured with CNTF as described above. The single labeled immunofluorescence assay was carried out after 3 and 7 days after neurogenic induction. The cells were firstly washed once with PBS and then fixed with 4% formalin (Sigma) at 4 °C for 10 min. The cells were permeabilized with 1% Triton-X-100 for 10 min and washed three times with PBS. Blocking solution containing 5% bovine serum albumin in PBS was added to the slides for 1 h. Primary antibodies were incubated with the slide at room temperature for 2 h and then secondary antibodies (1:500) were added for additional 1 h. Further wash step was carried out and DAPI was incubated with the slide for 30 min. The slides were washed twice and mounted prior to the analysis by fluorescence microscope (Leica, Germany). The primary antibodies used in this study were: rabbit anti nestin (1: 1000), CHAT (1:200), MAP 2 (1:200), and β-tubulin III (1:100) (Gene Tex, USA).

### Statistical analysis

All statistical calculations were performed with SPSSv.20.0 (SPSS Inc., Chicago, IL, USA) software. All data are expressed as mean ± standard deviation. Differences between groups were analyzed using analysis of variance (ANOVA) followed by *Dunnett’s test*, when appropriate. A *P* value of < 0.05 was considered statistically significant.

## Results

### SHED isolation and culture

**Fig. 1:**
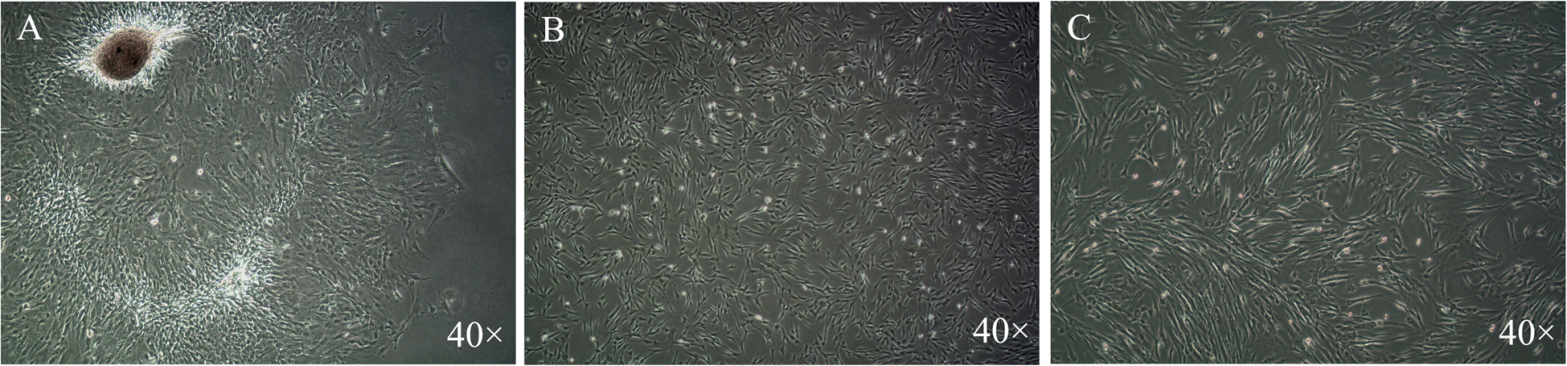
Primary stem cells from human exfoliated deciduous teeth of p0 (**a**), p1 (**b**) and p3 (**c**) passage. The cells exhibit uniform size and morphology with fibroblastic spindle shape (40x)

After enzymatic digestion, primary dental pulp tissues were implanted in the plate and separate cells around the tissues were observed 3 days after implantation. The cells were photorefractive and adherent to the wall. The cells around the tissue mass increased gradually, forming the appearance of cell colonies of halo 1 week after implantation (Fig. 1a). The shape of the cells was similar to that of fibroblasts (Fig. 1b). The cells were polygonal or fusiform, adherent to the wall well. The cells could be stably passed for up to 6 passages with consistent morphology and proliferation. P3 (Fig. 1c) cells were used in the following studies.

### Identification of SHEDs surface markers by flow cytometry

**Fig. 2:**
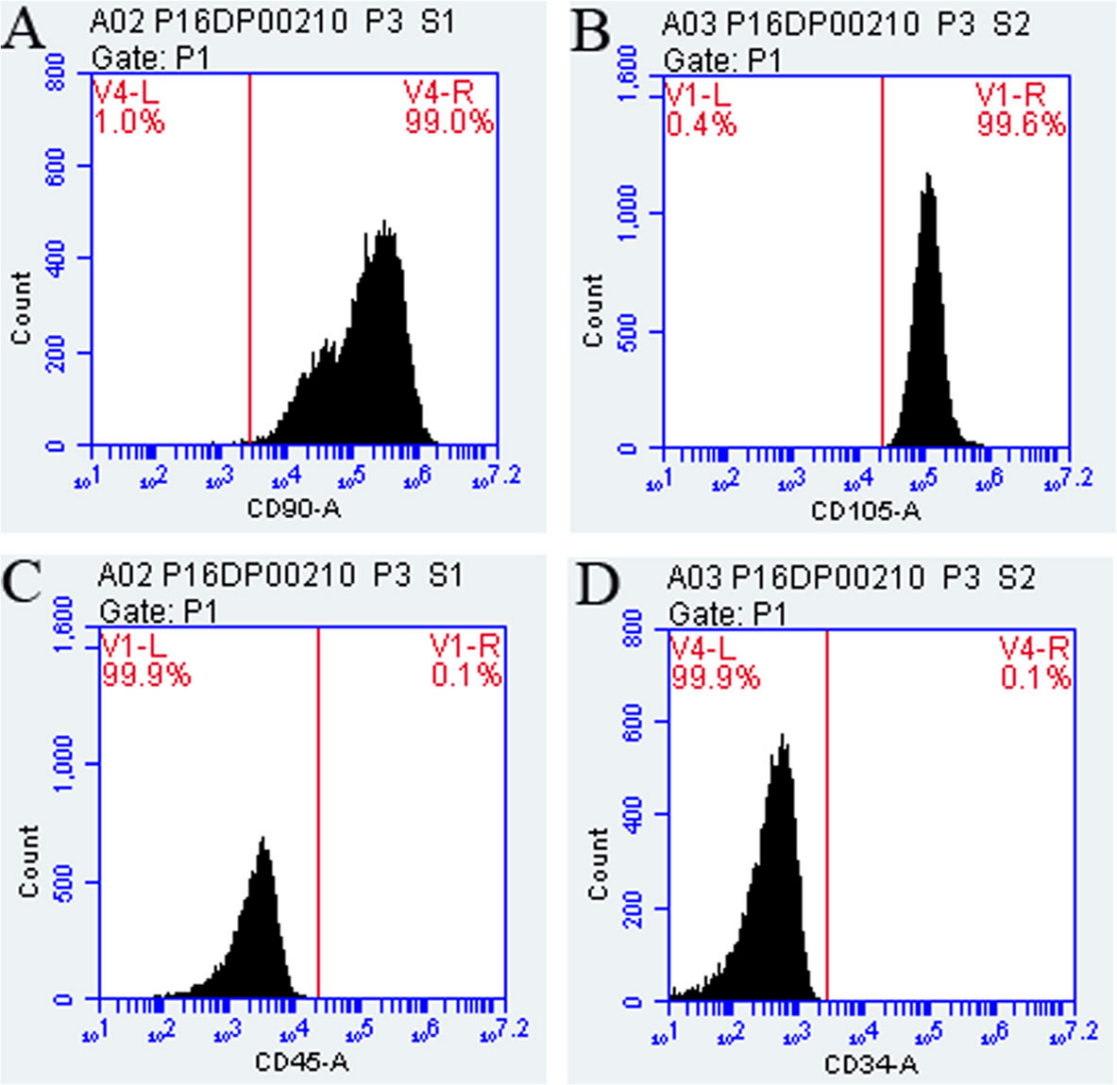
Expression of cell surface markers on SHEDs as determined by flow cytometry. The expression rate was 99.0, 99.6, 0.1, and 0.1% for CD90 (**a**), CD105 (**b**), CD45 (**c**), and CD34 (**d**), respectively

The identity of mesenchymal origin for the isolated SHEDs was determined. The results of flow cytometry indicated that the surface markers of mesenchymal stem cells CD90/CD105 were highly expressed in our isolated SHEDs, with an expression rate ≥ 95%. In contrast, these cells did not have a significant expression of CD34/CD45 (≤ 2%) which are surface markers for the stem cells of hematopoietic/endothelial origin (Fig. 2).

### Osteogenic and adipogenic differentiation of SHEDs

**Fig. 3:**
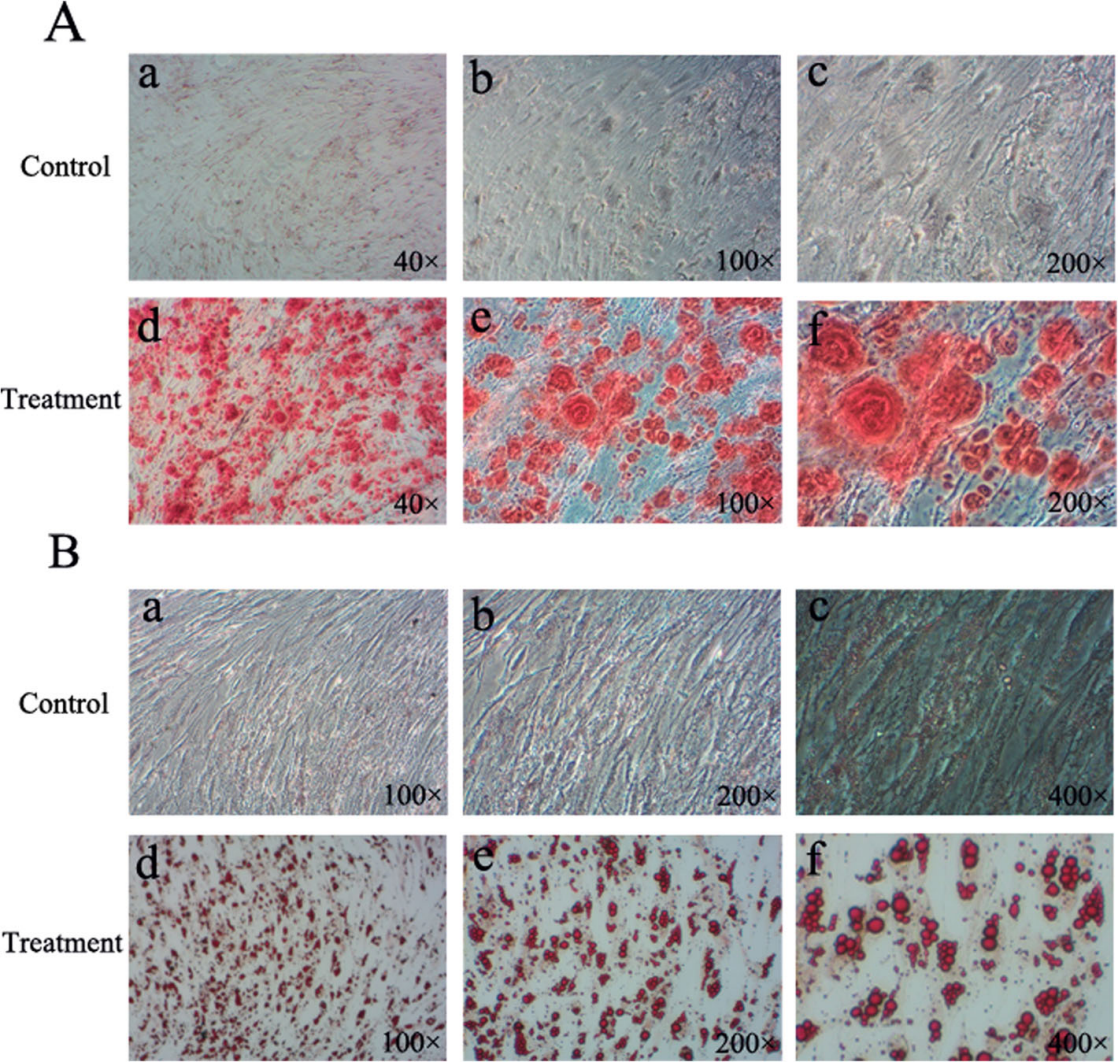
Osteogenic and adipogenic differentiation of SHEDs. **A**. Alizarin red staining for mineralized nodules in SHEDs in control (a: 40x; b: 100x; c: 200x) or osteogenic (d: 40x; e: 100x; f: 200x) medium. **B**. Oil red O staining for lipid droplets in SHEDs in control (a: 40x; b: 100x; c: 200x) or adipogenic (d: 40x; e: 100x; f: 200x) medium

To confirm the potential of multidirectional differentiation for the isolated SHEDs, we cultured SHEDs in osteogenic and adipogenic medium and examined the consequent mineralization and lipid accumulation in the cells. By using Alizarin red to stain calcium, we found that SHEDs cultured with osteogenic medium for 14 days had apparent formation of mineralized nodules as compared to those cultured in control medium which resulted in little staining (Fig. 3A). On the other hand, after induction with adipogenic medium for 3 weeks, Oil red O staining revealed that SHEDs generated much larger lipid vacuoles compared with the control (Fig. 3B).

### SHEDs morphology changes induced by neurogenic medium containing CNTF

**Fig. 4:**
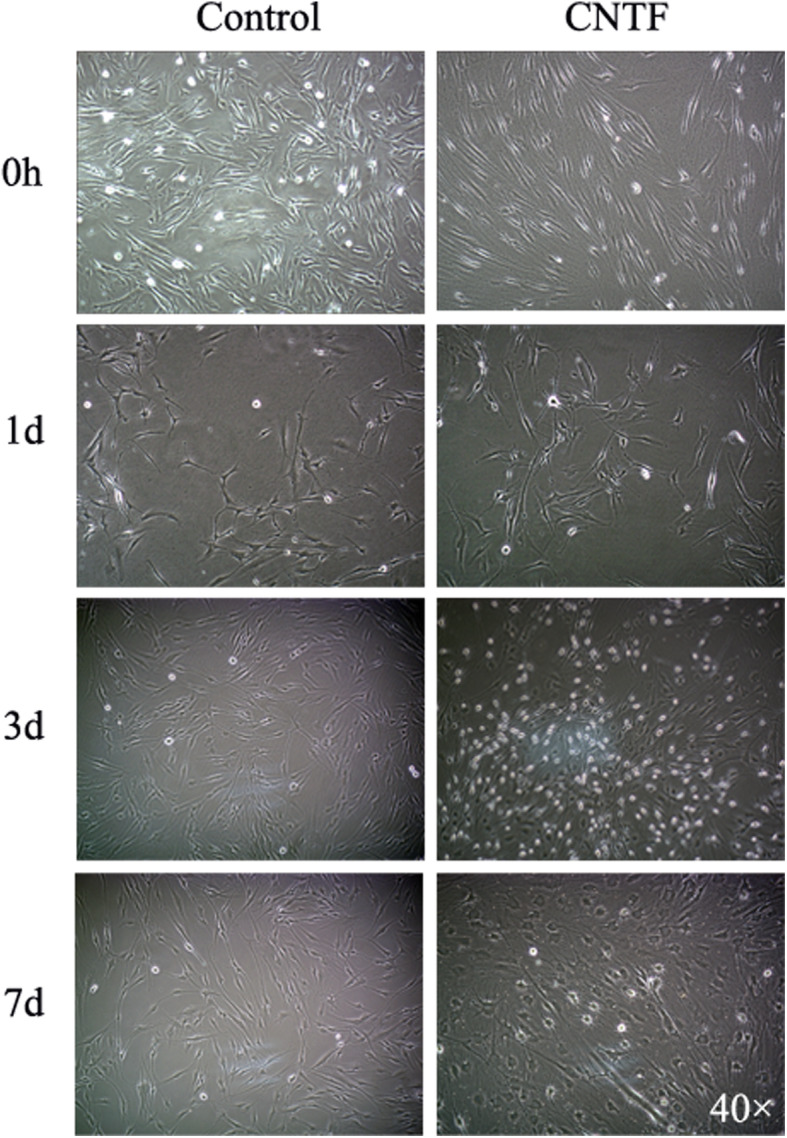
Effects of neurogenic medium and CNTF on SHED morphology. SHEDs were cultured in normal medium (0 h), neurogenic medium, or neurogenic medium containing 15 ng/L CNTF for 1, 3, and 7 days. The images were taken under light microscope (40x)

The undifferentiated SHEDs retained fibroblastic spindle shape. After 24 h of induction with neurogenic medium containing ciliary neurotrophic factor (15 ng/L), significant changes of cell morphology were observed under microscope (Fig. 4). The cytoplasm was constricted to the nucleus. Most of the cells were neuron-like cells with round cell bodies, long processes, and branches at the end of the processes. Some adjacent cells intertwined with each other. With longer times (3–7 days) of induction, more cells showed bipolar or multipolar neuron-like morphology, with structures similar to axons or dendrites and intertwined processes. The cell morphology became stable after 7 days of induction. The morphological changes seemed to be more dramatic for the cells cultured in the neurogenic medium containing CNTF as compared to those cultured without CNTF treatment, in particular at early culture times.

### Effect of CNTF on marker gene expression in neurogenic differentiation of SHEDs

**Fig. 5:**
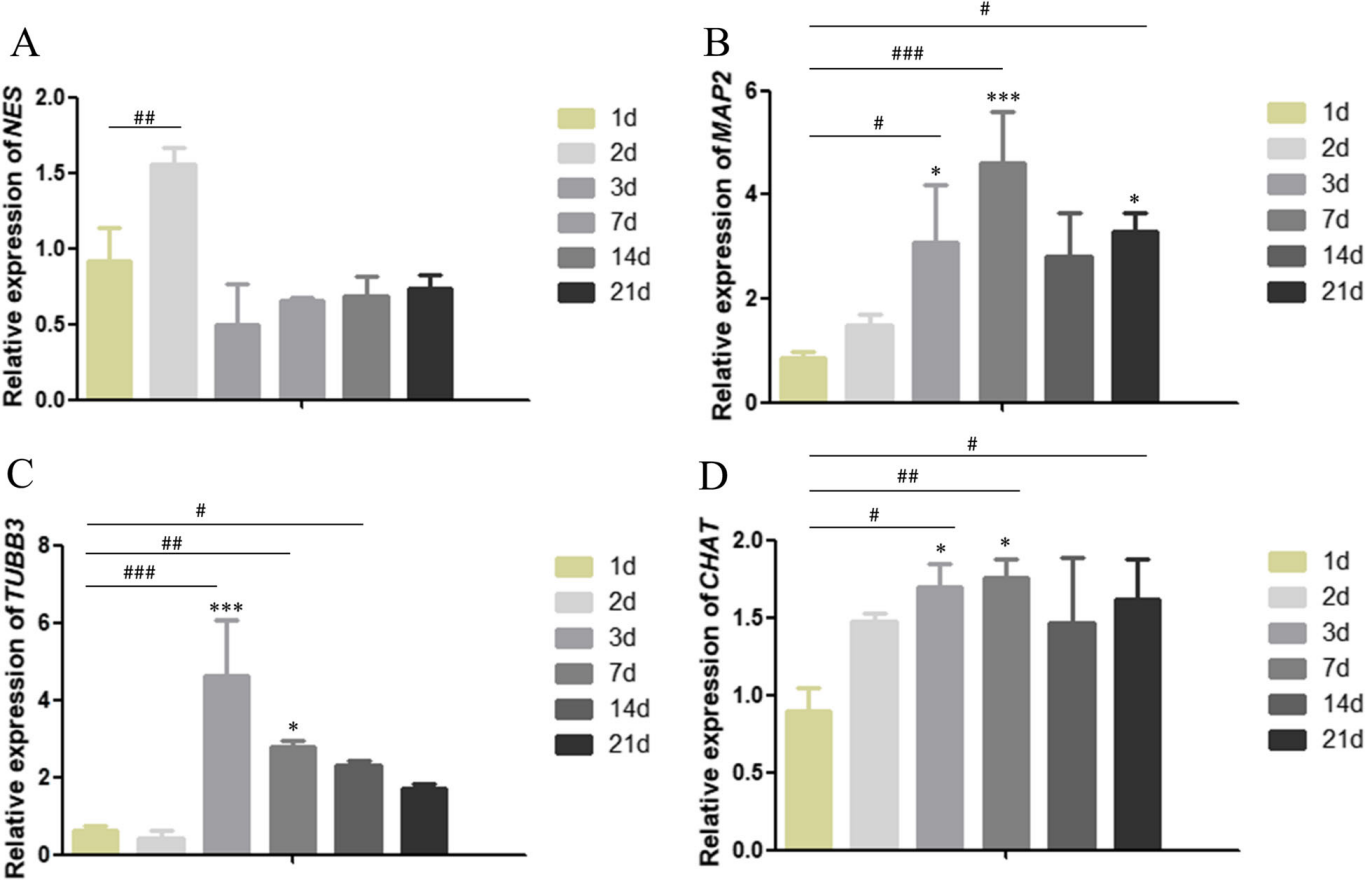
Effect of CNTF on marker gene expression in neurogenic differentiation of SHEDs. SHEDs were cultured in neurogenic medium without (control) or with CNTF (15 ng/L) for the indicated time. qRT-PCR was performed to examine the expression of *NES* (encoding Nestin) (**a**), *MAP 2* (**b**), *TUBB3* (encoding β-tubulin III) (**c**), and *CHAT* (encoding ChAT) (**d**) genes. Data are form 3 independent experiments in triplicate (*n* = 3). The data for each gene are normalized with the control on the same day. *p < 0.05, ** *p* < 0.01, ***p < 0.001 as compared with control on the same day. #*p* < 0.05, ##*p* < 0.01, ###*p* < 0.001 as compared with day 1

To determine the neurogenic potential of SHEDs, we chose to examine the expression of 4 marker genes over 21 days of induction (Fig. 5). Nestin is expressed in neuronal precursor cells. Upon differentiation, it becomes downregulated in neurons [29, 30]. In our studies, CNTF (15 ng/L) treatment led to a significant increase of nestin gene (*NES*) expression at day 2 as compared to the control of no CNTF treatment (Fig. 5a). However, the expression of nestin became decreased and stabilized afterwards, consistent with a differentiated status of SHEDs. MAP-2 plays an essential role in determining and stabilizing dendritic shape during neuron development [31, 32]. The expression of *MAP-2* gene was very low in undifferentiated SHEDs. It was dramatically induced by CNTF treatment (15 ng/L) at day 7 and maintained a slightly lower level afterwards (Fig. 5b). This expression pattern suggested that MAP-2 was mainly expressed in the protruding process of the neuron-like cells derived from SHED differentiation.

Tubulin is the main structure of neuronal microtubules. β-tubulin III is a type of tubulin that is involved in neuronal cell type-specific differentiation [31]. Similar to that of MAP-2, the expression of β-tubulin III gene (*TUBB3*) was significantly increased by CNTF treatment from day 7 to day 21 with the highest expression at day 7 (Fig. 5c). ChAT, an enzyme catalyzing the synthesis of acetylcholine, is a specific marker for cholinergic neurons. Interestingly, CNTF (15 ng/L) treatment led to a significant increase of *CHAT* gene expression since day 2 after induction (Fig. 5d).

### Effect of CNTF on marker protein expression in neurogenic differentiation of SHEDs

**Fig. 6:**
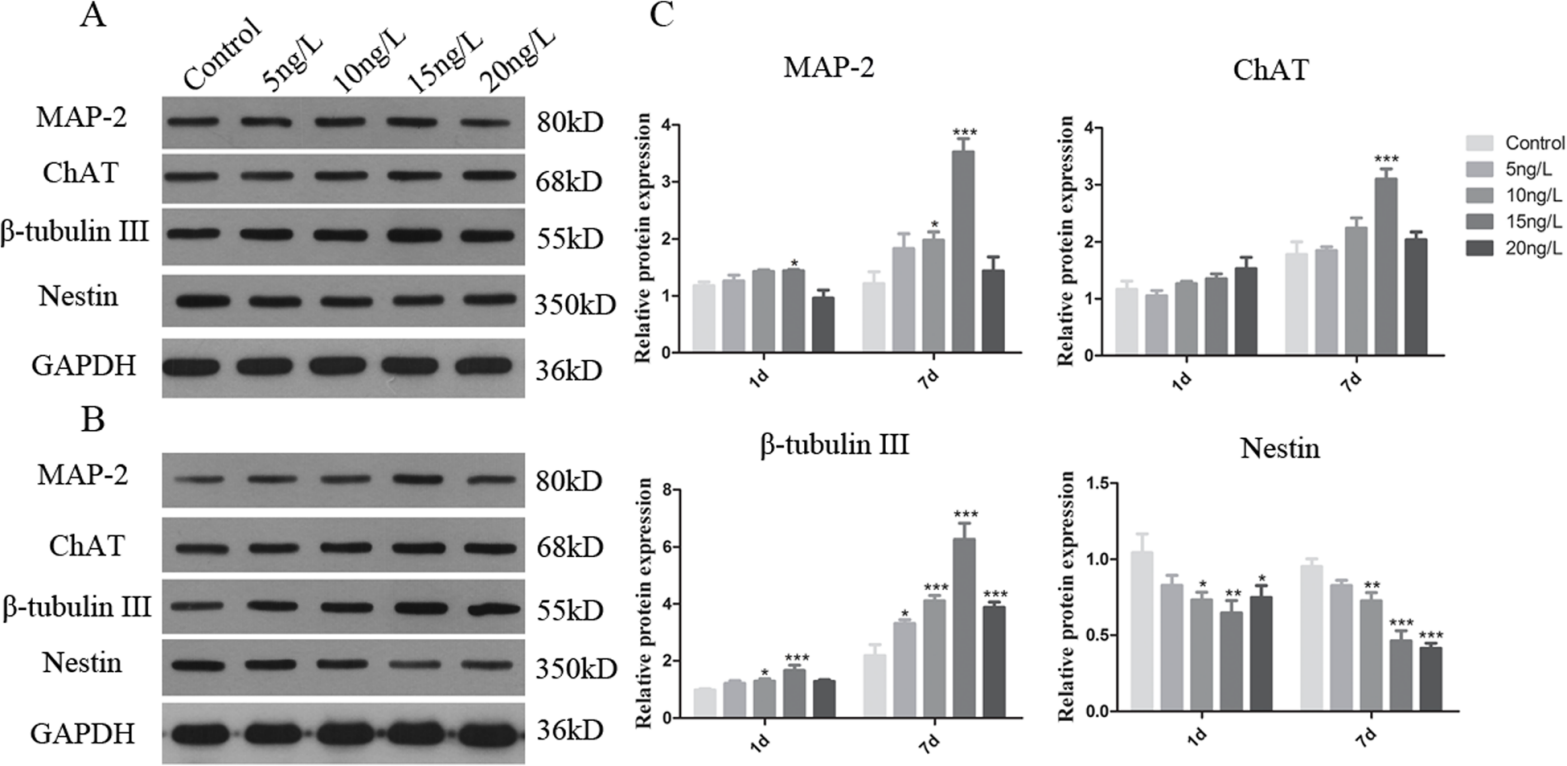
Effect of CNTF on marker protein expression during neurogenic differentiation of SHEDs. SHEDs were cultured in neurogenic medium with CNTF for one day (**a**) or seven days (**b**). The western blots were quantitated (C). GAPDH was used as the loading control. Data are form 3 independent experiments in triplicate (*n* = 3). *p < 0.05, **p < 0.01, ***p < 0.001 as compared with control

To validate the results of gene expression, we performed Western blotting to examine the effect of CNTF on the protein levels of the four markers. The protein expression for nestin, MAP 2, β-tubulin III, and ChAT is consistent with the transcript expression of the four corresponding genes respectively (Fig. 6 and Supplemental Fig. S1-S2). At 7th day after induction, the levels of MAP 2, β-tubulin III, and ChAT were all increased by CNTF treatment; however, the expression of nestin was little changed or even decreased over time as compared to the control (Fig. 6b-c and Supplemental Fig. S1–2).

**Fig. 7:**
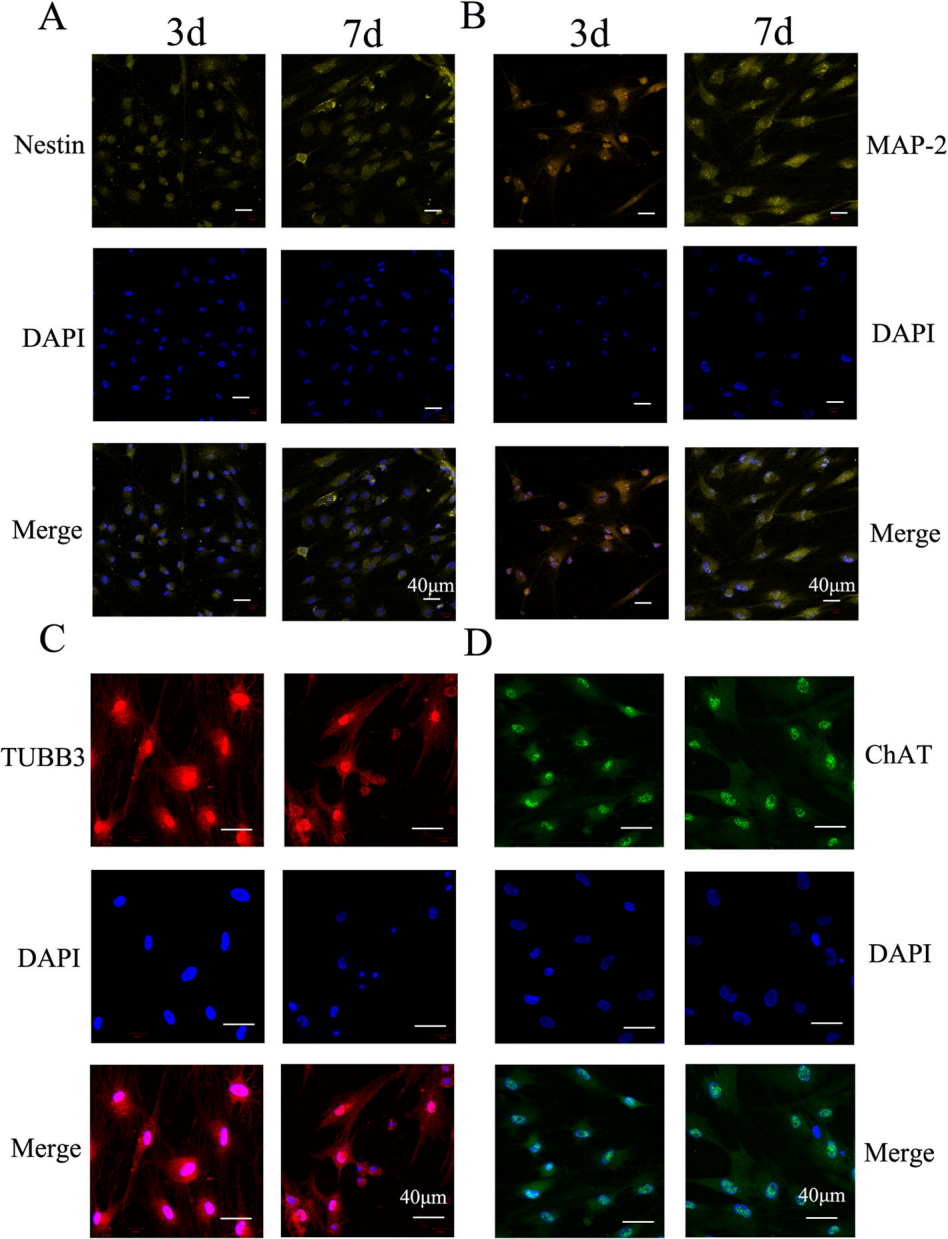
Immunofluorescence microscopy images showing the effect of CNTF on neurogenic differentiation of SHEDs. Four neurogenic marker proteins were studied: Nestin (**a**), MAP-2 (**b**), β-tubulin III (**c**), and ChAT (**d**). SHEDs were cultured in neurogenic medium with CNTF (15 ng/L) for 3 days or 7 days. Scale bar is 40 μm in all images

The expression of these marker proteins was further visualized by immunofluorescence microscopy that could provide the information of cellular location for proteins (Fig. 7 and Supplemental Fig. S3-S6). After 3 days of neurogenic induction by CNTF, we detected strong fluorescent signal of ChAT in cytoplasm and β-tubulin III in both cytoplasm and nucleus of SHEDs. With a longer induction by CNTF (15 ng/L), the expression of ChAT, β-tubulin III and MAP-2 remained at a high level up to 14 days. Both cytoplasm and nucleus expressed MAP-2. The expression of nestin was also consistently low after CNTF induction. Interestingly, the expression of the four marker proteins became lower after an even longer incubation with CNTF (15 ng/L) (Supplemental Fig. S3-S6), possibly related to a reduced proliferation after the long incubation.

Overall, the data of protein expression, together with those of gene expression data above, indicated that SHEDs had the potential to differentiate into neuro-like cells and specifically cholinergic neuro-like cells. The expression of nestin was decreased over culture time, while those of the other markers was maintained at a high level after neurogenic induction.

## Discussion

In this study, the SHEDs were isolated and cultured. The identity of mesenchymal origin and the multi-directional differentiation potential for these cells were validated. We found that CNTF treatment could facilitate neurogenic differentiation of SHEDs. In particular, with appropriate culture conditions and CNTF treatment, SHEDs was found to be able to differentiate into cholinergic neuro-like cells.

Treatment of nervous system diseases remains a major challenge in the clinic. The use of stem cells to replace, repair and strengthen damaged tissues and organs in order to achieve tissue regeneration and restore their function, or so called stem cell therapy, has become very attractive in recent years. Stem cell therapy requires stem cells to be readily available and high quality. The derivation and isolation of stem cells should be simple with minimal injury risk to the body. On the other hand, the stem cells should possess high stemness and the capability of differentiation into target cells and tissues. SHEDs are undoubtedly one of the best cell sources for stem cell therapy. They originate from the neural ridge of the ectoderm and belong to mesenchymal stem cells. The SHEDs are isolated from the deciduous teeth that are about to be replaced, easy to obtain, and essentially non-invasive. The ethical controversy thus could be much less than those for other types of stem cells such as embryonic stem cells. The SHEDs have a high proliferative activity with a doubling time that is reported to be shorter than DPSCs and BMSCs [33]. In particular, it has been reported that the SHEDs express surface markers of neurons such as Nestin and β-tubulin III [34], suggesting their potential application in the neurological field.

It has been reported that dental pulp stem cells can provide neurotrophic support for dopaminergic neurons and differentiate into neurons [35]. After implantation, they might secrete nerve growth factors that could act on dopaminergic neurons, thus promoting the recovery of injured dopaminergic neurons. SHEDs have been reported to be able to differentiate into nerve cells under certain induction conditions, and their transplantation has a therapeutic effect in rat Parkinson’s disease model. As glial cell-derived neurotrophic factors are recognized to promote the survival of midbrain dopaminergic cells, a possible mechanisms is to protect dopaminergic cells by secreting such factors [13]. Morsczeek reported the culture of SHEDs in different serum-replacement media (SRM) [36]. SHEDs formed neurosphere-like cell clusters in SRM with the B27 supplement, epidermal growth factor, and fibroblast growth factor-2. Moreover, the glial cell marker glial fibrillary acidic protein was significantly expressed. After neural-directional induction, SHEDs lost the original morphology of fibroblasts. These findings indicated that SHEDs can be differentiated into specific type of neuron-like cells with optimized culture conditions.

CNTF is a very effective neurotrophic factor that can promote neural regeneration [37]. While a toxic treatment can cause the death of cultured spinal motor neurons, CNTF has a significant rescuing effect on the survival of injured spinal cord neurons [38]. CNTF can also prevent the degenerative loss of neurons in vivo and the degenerative changes caused by axonal transection of facial neurons in newborn mice [39]. CNTF may even promote axonal regeneration of motor neurons [40]. Because of the widespread distribution of CNTF in the brain and its presence in glial cells, CNTF may also act as a protective factor for glial cells [41]. However, CNTF is not only a neurotrophic factor, it also acts as a cholinergic differentiation factor for sympathetic neurons. It has been reported that CNTF can reduce the level of tyrosine hydroxylase (TH) by increasing ChAT and transform adrenal neurons into cholinergic neurons [39].

Huang et al. found that CNTF, brain-derived neurotrophic factor (BDNF) and their combination could induce the differentiation of human umbilical cord blood mesenchymal stem cells into neuron-like cells in vitro [42]*.* Zeng et al. reported that CNTF together with salvia miltiorrhiza could induce differentiation of myogenic stem cells into neuron-like cells [19]. In the present study, we confirmed for the first time that CNTF could promote the differentiation of SHEDs into cholinergic neuron-like cells. ChAT serves a specific marker for cholinergic neurons. The immunoreactivity of ChAT is often used as a marker of cognitive decline in various neurodegenerative diseases such as Alzheimer’s disease (AD). We found that SHEDs did not express ChAT without induction. After neurogenic induction, particularly with CNTF treatment, ChAT was highly expressed in the nucleus and cytoplasm of the induced neuron-like cells. The effect by CNTF was dependent on its concentration and treatment time. Like many other studies [43, 44], we have focused on and observed the expression of specific markers in the induced neuron-like cells. Of note, however, our study has limitation as we have yet to examine the neural function of those induced neuron-like cells. In future studies, it is important to further explore the most optimal culture conditions with CNTF and characterize the neural function in order to employ SHEDs in cell therapy to treat neurological diseases such as AD.

In conclusion, our study confirmed that CNTF can promote the differentiation of SHEDs into cholinergic neuron-like cells. The findings have laid a foundation for the further study of cell therapy using SHEDs in the field of neurological diseases.

## Supplementary Information

**Additional file 1: Figure S1.** Dose and treatment time dependent effect of CNTF on neurogenic marker protein expression. SHEDs were cultured in DMEM medium (Blank) or neurogenic medium without (control) or with different concentrations of CNTF (5–20 ng/L) as indicated for 1 day (A), 3 days (B), 7 days (C) or 14 days (D). **Figure S2.** Treatment time dependent effect of CNTF (15 ng/L) on neurogenic marker protein expression in SHEDs. **Figure S3.** Immunofluorescence microscopy images showing the effect of CNTF (15 ng/L) on nestin of SHEDs for 1 day, 3 days, 7 days, 14 days or 21 days. Scale bar is 40 μm in all images. **Figure S4.** Immunofluorescence microscopy images showing the effect of CNTF (15 ng/L) on MAP-2 of SHEDs for 1 day, 3 days, 7 days, 14 days or 21 days. Scale bar is 40 μm in all images. **Figure S5.** Immunofluorescence microscopy images showing the effect of CNTF (15 ng/L) on β-tubulin III of SHEDs for 1 day, 3 days, 7 days, 14 days or 21 days. Scale bar is 40 μm in all images. **Figure S6.** Immunofluorescence microscopy images showing the effect of CNTF (15 ng/L) on ChAT of SHEDs for 1 day, 3 days, 7 days, 14 days or 21 days. Scale bar is 40 μm in all images.

## Abbreviations

SHEDs: Stem cells of human exfoliated deciduous teeth
CNTF: Ciliary neurotrophic factor
CHAT: Acetylcholine transferase
MAP 2: Microtubule-associated protein 2
DPSCs: Dental pulp stem cells
CNC: Cranial neural crest
BMSCs: Bone marrow stem cells
SRM: Serum-replacement media
TH: Tyrosine hydroxylase
BDNF: Brain-derived neurotrophic factor
AD: Alzheimer’s disease
