# Supplementary material for: Effect of ciliary neurotrophic factor on neural differentiation of stem cells of human exfoliated deciduous teeth
Source: J Biol Eng. 2020 Dec 9;14:29. doi: 10.1186/s13036-020-00251-4 (PMC7724848; doi:10.1186/s13036-020-00251-4)
Supplement: Supplementary file 1 — Additional file 1: Figure S1. Dose and treatment time dependent effect of CNTF on neurogenic marker protein expression. SHEDs were cultured in DMEM medium (Blank) or neurogenic medium without (control) or with different concentrations of CNTF (5–20 ng/L) as indicated for 1 day (A), 3 days (B), 7 days (C) or 14 days (D). Figure S2. Treatment time dependent effect of CNTF (15 ng/L) on neurogenic marker protein expression in SHEDs. Figure S3. Immunofluorescence microscopy images showing the effect of CNTF (15 ng/L) on nestin of SHEDs for 1 day, 3 days, 7 days, 14 days or 21 days. Scale bar is 40 μm in all images. Figure S4. Immunofluorescence microscopy images showing the effect of CNTF (15 ng/L) on MAP-2 of SHEDs for 1 day, 3 days, 7 days, 14 days or 21 days. Scale bar is 40 μm in all images. Figure S5. Immunofluorescence microscopy images showing the effect of CNTF (15 ng/L) on β-tubulin III of SHEDs for 1 day, 3 days, 7 days, 14 days or 21 days. Scale bar is 40 μm in all images. Figure S6. Immunofluorescence microscopy images showing the effect of CNTF (15 ng/L) on ChAT of SHEDs for 1 day, 3 days, 7 days, 14 days or 21 days. Scale bar is 40 μm in all images. [file 13036_2020_251_MOESM1_ESM.docx]

**Supplementary Materials Legends**

**
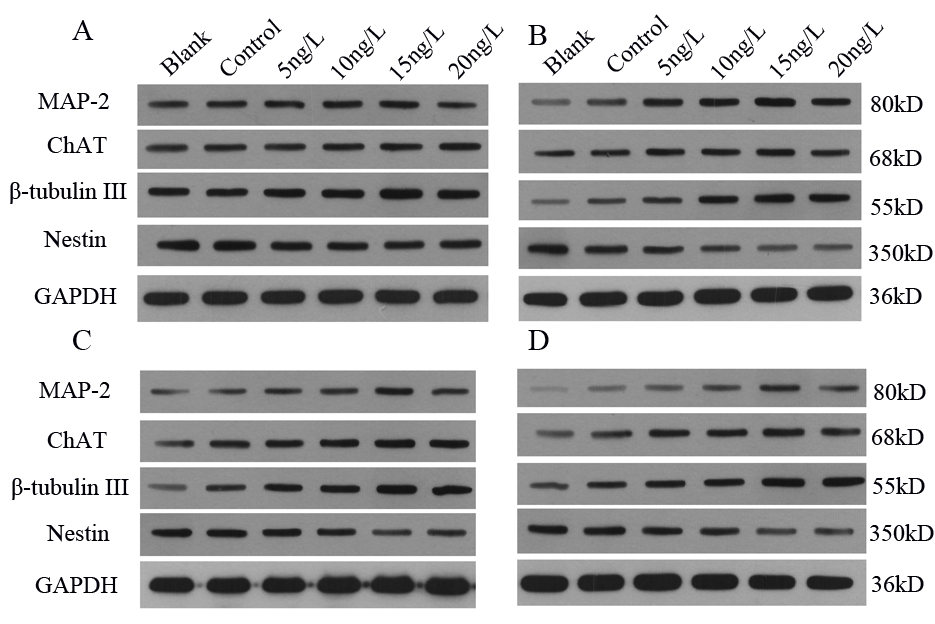
**

**Additional file: Figure S1. Dose and treatment time dependent effect of CNTF on neurogenic marker protein expression.** SHEDs were cultured in DMEM medium (Blank) or neurogenic medium without (control) or with different concentrations of CNTF (5 - 20 ng/L) as indicated for 1 day (A), 3 days (B), 7 days (C) or 14 days (D).


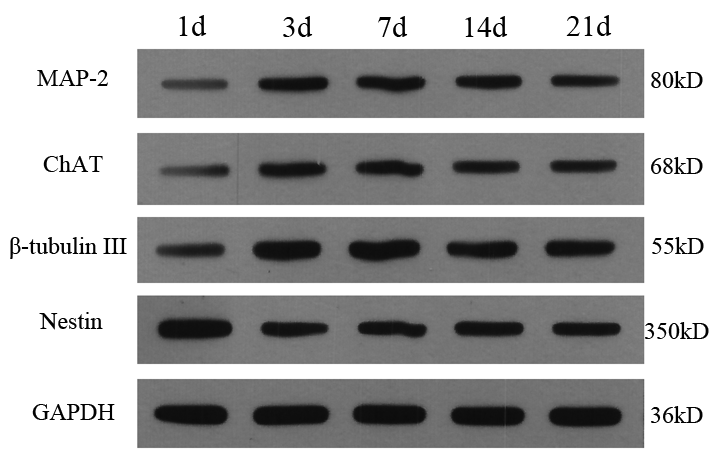


**Additional file: Figure S2. Treatment time dependent effect of CNTF (15ng/L) on neurogenic marker protein expression in SHEDs.**

**
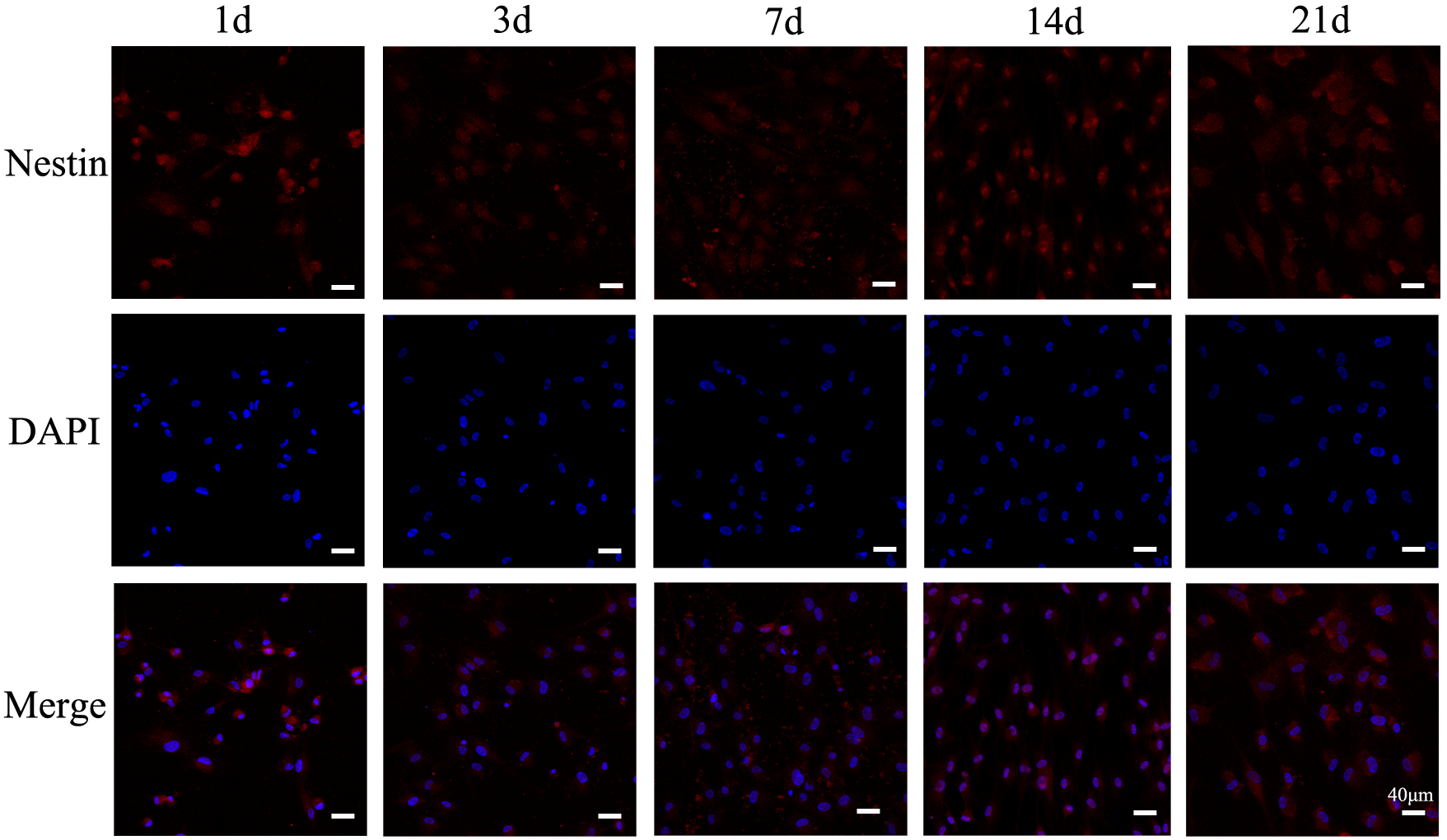
**

**Additional file: Figure S3. Immunofluorescence microscopy images showing the effect of CNTF (15ng/L) on nestin of SHEDs** **for** **1 day, 3 days, 7 days, 14 days or 21 days.** Scale bar is 40 μm in all images.


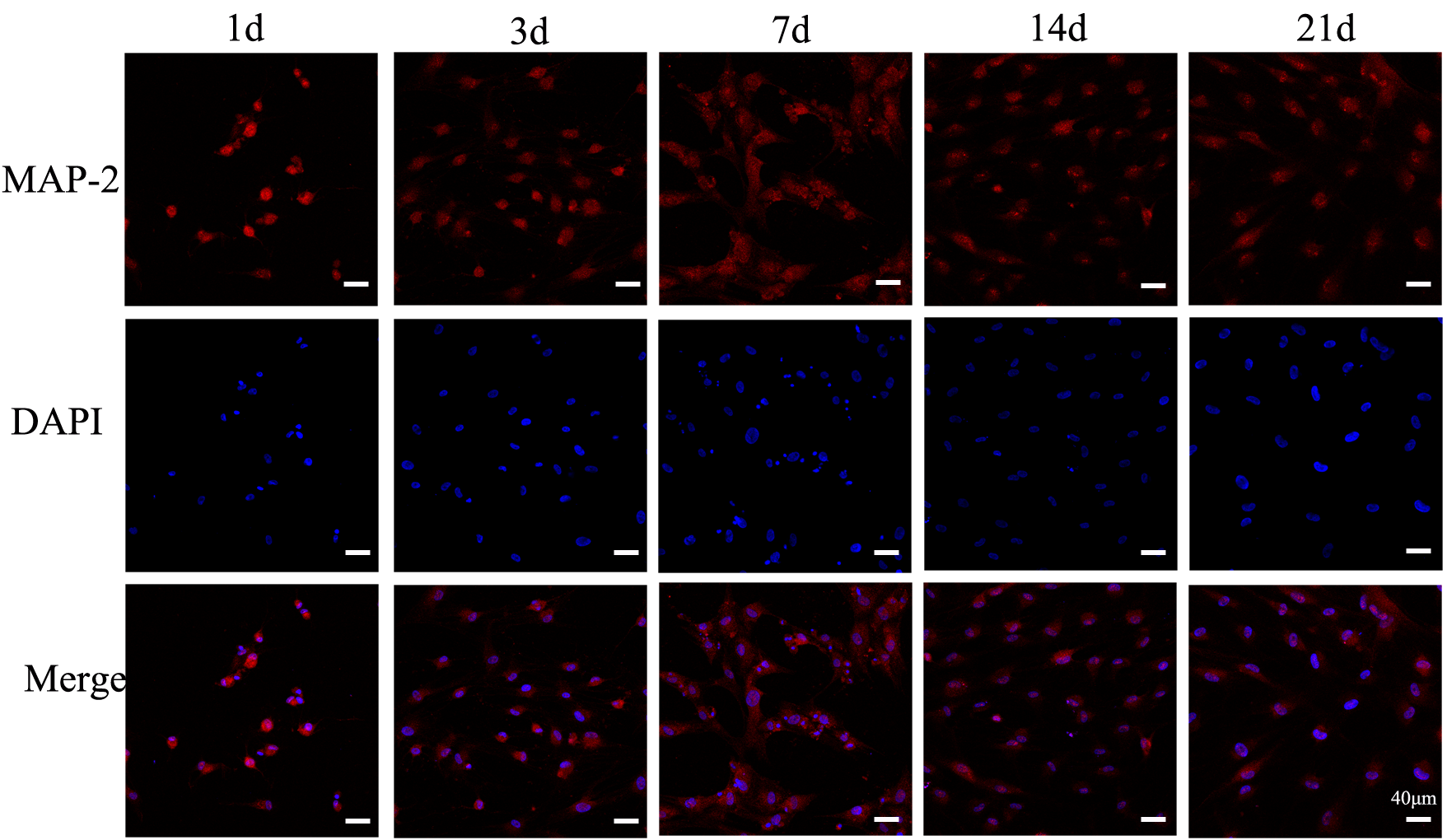


**Additional file: Figure S4. Immunofluorescence microscopy images showing the effect of CNTF (15ng/L) on MAP-2 of SHEDs for 1 day, 3 days, 7 days, 14 days or 21 days.** Scale bar is 40 μm in all images.


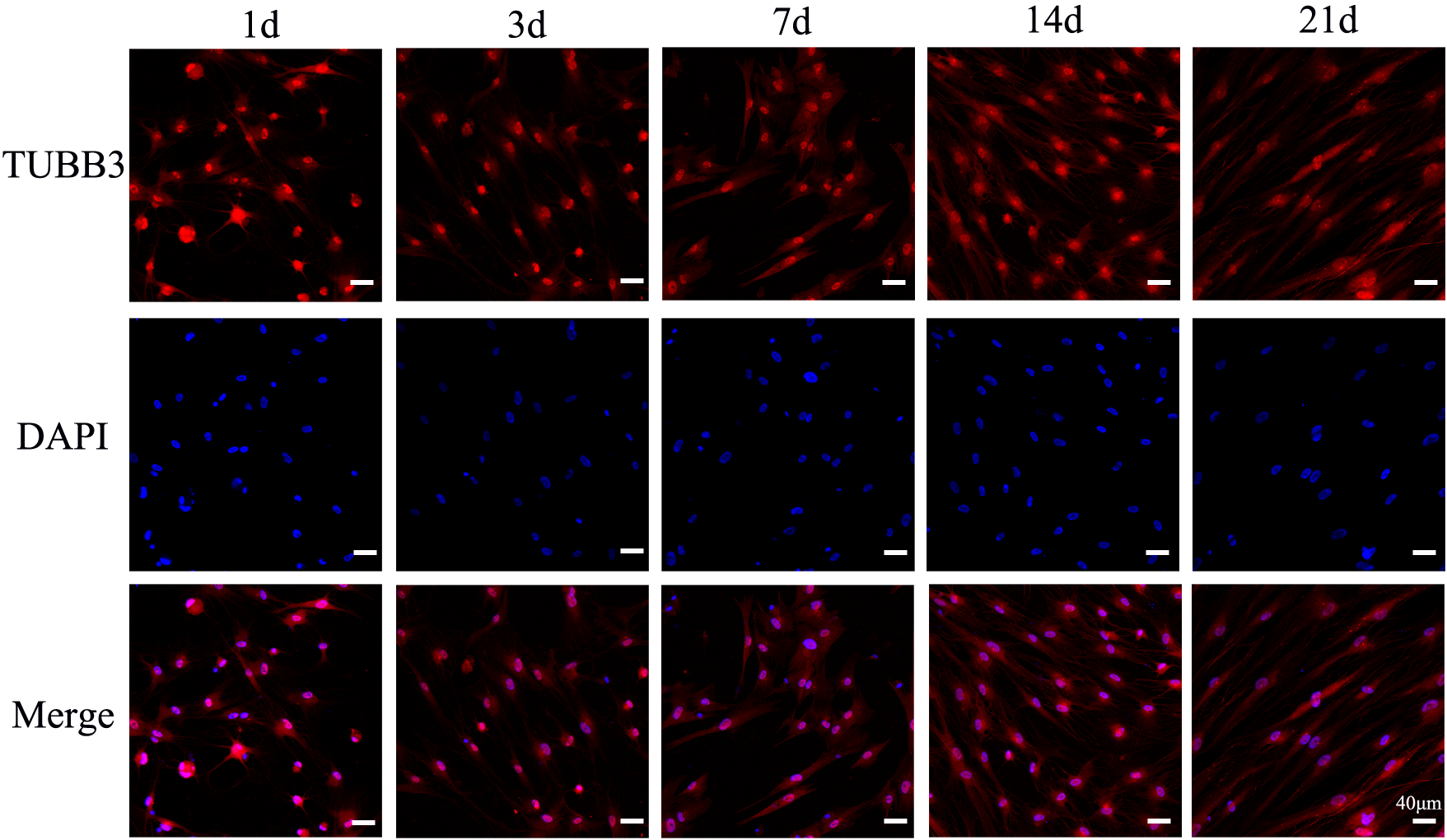


**Additional file: Figure S5. Immunofluorescence microscopy images showing the effect of CNTF (15ng/L) on β-tubulin III of SHEDs for 1 day, 3 days, 7 days, 14 days or 21 days.** Scale bar is 40 μm in all images.


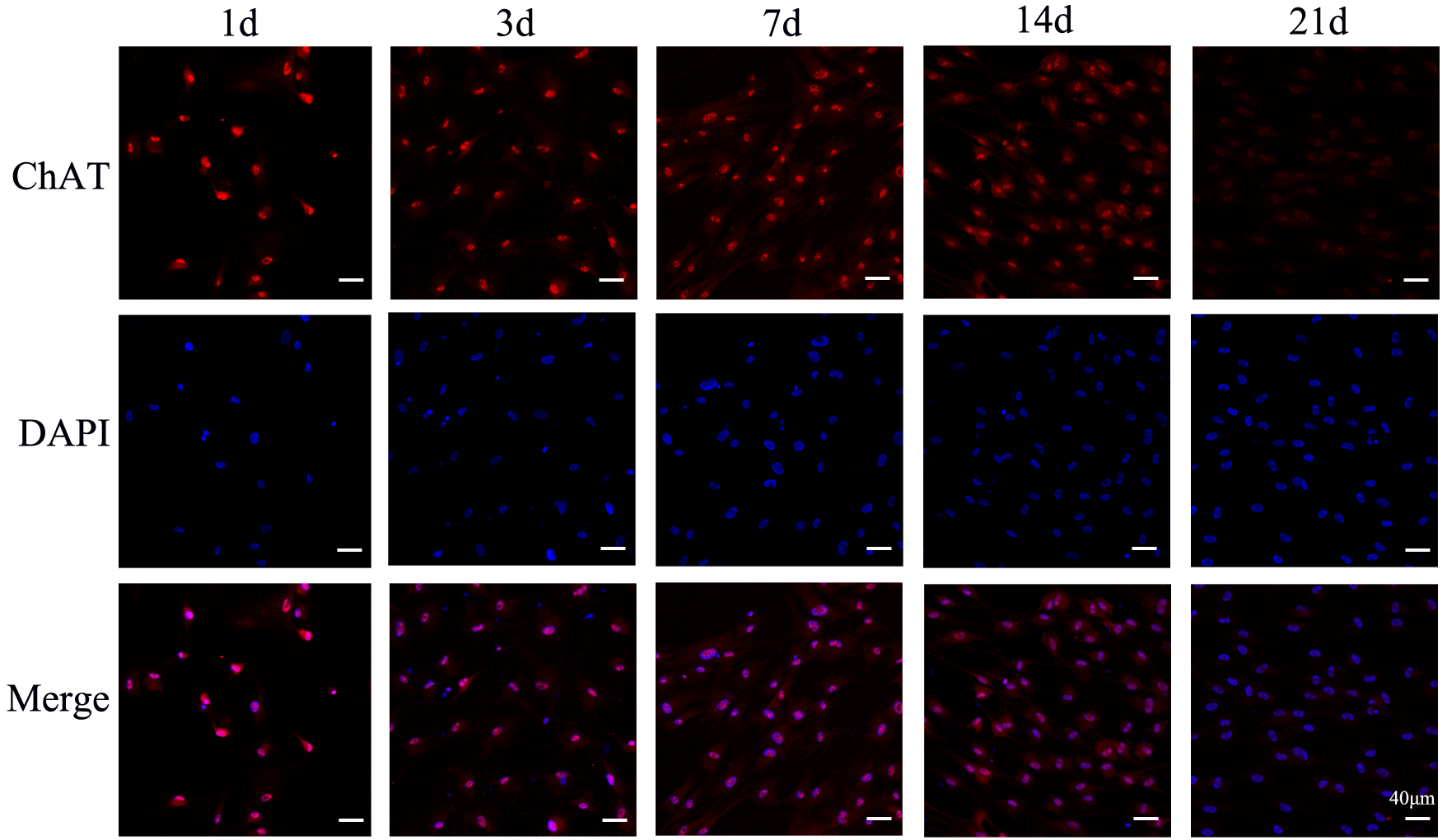


**Additional file: Figure S6. Immunofluorescence microscopy images showing the effect of CNTF (15ng/L) on ChAT of SHEDs for 1 day, 3 days, 7 days, 14 days or 21 days.** Scale bar is 40 μm in all images.
